# The Role of Urban/Rural Environments on Mexican Children’s Connection to Nature and Pro-environmental Behavior

**DOI:** 10.3389/fpsyg.2020.00514

**Published:** 2020-03-20

**Authors:** Maria Fernanda Duron-Ramos, Silvia Collado, Fernanda Inéz García-Vázquez, Maria Bello-Echeverria

**Affiliations:** ^1^Department of Psychology, Instituto Tecnológico de Sonora, Guaymas, Mexico; ^2^Department of Psychology and Sociology, Faculty of Economics and Business, Universidad de Zaragoza, Zaragoza, Spain; ^3^Department of Education, Instituto Tecnológico de Sonora, Ciudad Obregón, Mexico; ^4^Department of Postgraduate, Universidad de Sonora, Hermosillo, Mexico

**Keywords:** connection, nature, pro-environmental behavior, urban, rural

## Abstract

Living in rural areas has been described a driver for behaving in a pro-environmental way, mainly due to the more frequent contact with nature that people from rural areas have. However, the processes that link living in a rural area and behaving in a more ecological manner have not been systematically studied. Moreover, most studies have focused on adults living in developed countries. Given the importance that the actions conducted by people in developing countries have for the future of the environment, as well as the relevance of children’s pro-environmentalism for nature conservation, we present a brief research report examining the relationship between Mexican children’s place of residence and self-reported pro-environmental behavior (PEB). Participants were 200 children from Mexican rural areas (<1,000 inhabitants) and 200 from a Mexican urban city (>150,000 inhabitants). Children were between 9 and 12 years old. Children’s connection to nature was considered as a mediator in the relationship between children’s place of residence and PEB. Our findings revealed that rural children hold a stronger sense of connection to nature and behave in a more pro-environmental way than urban children. In addition, place of residence was directly and positively linked to their PEBs, and this relationship was mediated by children’s connection to nature. The relationship between connection to nature and PEB was stronger for girls than for boys. The model explained 45% of the variance of children’s self-reported PEBs.

## Introduction

Human actions negatively affect the health of our planet (Milfont and Schultz, 2016; Evans, 2019). Environmental psychologists have long tried to find ways to mitigate the negative consequences that human behavior has on nature, mainly through the promotion of a sustainable way of living (Schutte and Bhullar, 2017; Rosa et al., 2018). One way of doing this is through instilling pro-environmental behaviors (PEBs). PEB have been described as deliberate and effective behaviors that protect the natural environment (Corral, 2010).

Most of the studies of PEB have been conducted with adults. The role played by children in the protection of nature has been largely overlooked (Collado and Sorrel, 2019). Moreover, the majority of knowledge gained about the drivers of PEB relates to people living in developed countries. This ignores those living in developing countries who, according to the World Trade Organization (World Trade Organization [WTO], 2014), have an increasing impact on the health of the planet. Given this, we believe it is relevant to examine the factors and processes leading to the PEB of children from developing countries.

According to previous studies, some of the drivers of children’s PEB include frequent contact with nature (Evans et al., 2018; Collado and Evans, 2019; Otto et al., 2019), pro-environmental attitudes (Cheng and Monroe, 2012; Larson et al., 2015), social norms (Casaló and Escario, 2016; Evans et al., 2018), and perceived restorativeness (Collado and Corraliza, 2015). To the best of our knowledge, there has been little research done on the role played by the child’s environment on their PEB, especially in developing countries. In the current study, we investigate whether Mexican children’s place of residence (urban/rural) is linked to their PEB, and if this relationship is mediated by children’s sense of emotional connection to nature (Mayer and Frantz, 2004).

### Place of Residence, Connection to Nature, and PEB

Rural residents spend more time in nature than their urban counterparts (Gifford and Nilsson, 2014), and tend to recall experiences in the natural environment as positive (Chawla and Derr, 2012). This pattern holds both for adults and children (Lekies and Brensinger, 2017). In line with previous researchers (Hinds and Sparks, 2008; Gifford and Nilsson, 2014), this study assumes that children living in rural areas have more frequent contact with nature than those living in urban ones. Pleasant experiences in nature lead to increased environmental responsibility (Berenguer et al., 2005; Evans et al., 2018) and connection to nature (Rosa et al., 2019). However, the pathways to this relation are unknown, especially among children from developing countries (Bratman et al., 2019). The present study considers connection to nature as a possible mediator of the relationship between those living in an urban and those in a rural context as well as PEB in Mexican children.

A greater connection to nature often leads to higher interest in taking care of the natural resources (Nisbet et al., 2008) and more frequent PEB (Schultz, 2001; Mayer and Frantz, 2004; Olivos et al., 2013). Of interest to the current study, Hinds and Sparks (2008) found that living in a rural area as a child promotes connection to nature which, in turn, leads to more frequent PEB in adulthood. Collado et al. (2015) concluded that children who live in rural areas show stronger environmental attitudes and connection to nature which, in turn, lead to children’s PEB. The relationship between children’s environmental attitudes and PEB differed according to children’s place of residence, which determined the amount of time children spent in nature. Similarly, De Dominicis et al. (2017) found that the effect of participating in an environmental education program organized in a natural environment on children’s PEB differed according to children’s place of residence. According to the authors, rural children spend more time in nature than urban ones. This leads rural children to behave in a more pro-environmental way, and might be the reason why the environmental education program is less effective for them.

### The Present Study

Given the scarcity of studies of the determinants of children’s PEB, especially in developing countries, we focus on the study of the relationship between urban/rural residency of Mexican children and their PEB. We also evaluate whether connection to nature mediates the relationship between children’s place of residence and PEB. This specific sample was chosen for two primary reasons. First, Mexico is a developing country and has a large biodiversity within its territory, which needs to be preserved (Calderon-Aguilera et al., 2012). Second, in contrast to children from urban areas, rural children in Mexico live in direct contact with nature (Vargas, 2010).

We expect the children’s place of residence (urban/rural) to be linked to their PEB. Specifically, children from rural areas are expected to show stronger PEB than those from urban ones (Hypothesis 1, H1). Children’s CN is expected to mediate the relation between children’s place of residence and PEB (Hypothesis 2, H2). Women (Gifford and Nilsson, 2014) and girls (Duarte et al., 2017) tend to report higher PEBs than men and boys. One reason for this is that females endorse higher environmental attitudes (Duerden and Witt, 2010) and emotional empathy (Arnocky and Stroink, 2010) than men, which usually lead to more frequent PEB. Additionally, findings from previous studies suggest that the association between PEB and its determinants varies from boys to girls (Collado et al., 2015). Considering this, we explored the possible variations in the direct and indirect associations between the place of residence and PEB according to gender (i.e., moderating role of gender), without any specific hypothesis in mind.

## Materials and Methods

### Participants

Four hundred children from 9 to 12 years old (*M* = 10, *SD* = 0.73) participated in the study. Half of them lived in rural and indigenous communities (i.e., <1,000 inhabitants) in Northern Mexico. The rest lived in an urban area with >150,000 inhabitants. Fifty-four percent of the participants were girls.

### Measures

#### Place of Residence

Place of residence was coded as 1 (urban) and 2 (rural).

#### Self-Reported PEB

Pro-environmental behavior was recorded using the general ecological behavior scale of Kaiser (1998), adapted for use with children by Fraijo et al. (2012). This instrument includes 15 items related to PEB, such as reuse, recycle, as well as energy conservation. For example, “When performing a school project, I try to reuse material.” Responses were rated using a scale from 0 (never) to 3 (always). α = 0.78.

#### Connection to Nature

Connection to nature was registered using the children’s affective attitude toward nature scale (Cheng and Monroe, 2012). This instrument is formed by 17 items (e.g., “Humans are part of the natural world”) and responses used a scale from 1 (strongly disagree) to 5 (strongly agree). α = 0.84.

#### Gender

Gender was coded as 1 (boys) and 2 (girls).

### Procedure

The study was approved by the Technological Institute of Sonora (Mexico). Fifty schools were invited to participate and 12 of them agreed. Participations were restricted to children with a written authorization from their parents. Paper-and-pencil questionnaires were completed individually at school with assurance of anonymity. Data collection took about 30 min.

#### Data Analysis

First, descriptive, correlational, and *t*-tests analyses were conducted. Then, a mediational model was carried out using PROCESS (Hayes, 2018), model 14.^1^ This particular model provides the direct relation between place of residence (urban/rural) and PEB (H1). It also estimates the indirect effect on the dependent variable (PEB) through the connection to nature (H2), as well as the possible moderating role of gender.

## Results

Children from both urban and rural places of residence show a high sense of connection to nature, being higher for those living in rural areas (*t* = 360.76, *p* < 0.00). However, they report a low frequency of conducting PEB, with urban children reporting a lower frequency than rural children (*t* = 395.13, *p* < 0.00). Girls reported more connection to nature (*t* = 358.60, *p* < 0.00) than boys, while PEB is very similar for both genders. We found a moderate positive correlation (Cohen, 1988) between children’s place of residence and PEB, as well as between connection to nature and PEB (Table 1).

**TABLE 1:** Descriptive statistics and correlation matrix.

	Descriptive statistics				Descriptive statistics				Correlation matrix		
									Place of	Connection to	
	Mean	SD	Mean	SD	Mean	SD	Mean	SD	residence	nature	PEB
	Urban		Rural		Girls		Boys		1		
Connection to nature	4.25	0.56	4.45	0.40	4.51	0.40	4.12	0.56	0.20**	1	
PEB	1.53	0.52	2.18	0.48	1.98	0.56	1.57	0.63	0.55**	0.46**	1

**p < 0.01; PEB, pro-environmental behavior.

**FIGURE 1:**
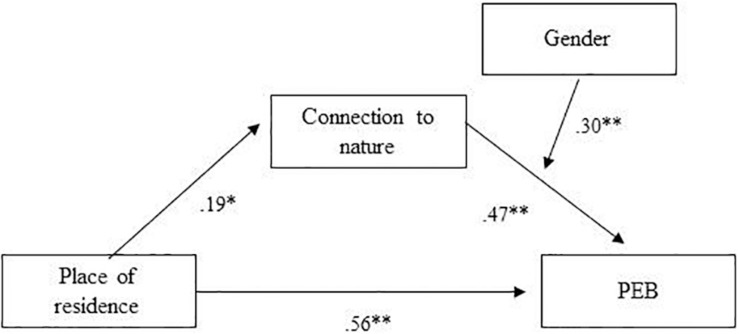
Results for the multiple mediator regression model (*n* = 400). All path coefficients are unstandardized beta values. *p < 0.01, **p = 0.001. PEB, self-reported pro-environmental behavior (R^2^ = 0.45).

The mediating model shows a positive, direct link between place of residence (urban/rural) and children’s PEB [β = 0.56, 95% CI (0.47, 0.65)]. We also found an indirect relationship between place of residence and PEB mediated by connection to nature [β = 0.19, 95% CI (0.09, 0.28)]. Gender (boy/girl) was found to moderate the link between connection to nature and PEB [β = 0.30, 95% CI (0.12, 0.48)], with the relation between connection to nature and PEB being stronger for girls than for boys. *R*^2^ for PEB was 0.45 (Figure 1).

## Discussion

The growing visibility of environmental damage has led to an increase of environmental concern (Arı and Yılmaz, 2016). Consequently, the number of studies examining the factors and processes leading to PEB has also increased. However, little is known about the pathways to children’s pro-environmentalism (Otto et al., 2019), especially in developing countries. To fill this gap in the literature, we evaluated the role that place of residence (urban/rural) plays in Mexican children’s PEB. We also checked whether connection to nature is behind the link between place of residence and PEB, and explored the possible moderating role of gender.

According to our results, living in rural areas seems to be positively linked with children’s frequency of PEB (H1). This is in line with previous studies (Cheng and Monroe, 2012; Rosa et al., 2019) which demonstrate that time spent in rural areas, and hence in contact with nature (Gifford and Nilsson, 2014), is one of the main determinants of people’s PEB. In contrast to previous studies conducted in developed countries (Cheng and Monroe, 2012), our study shows that Mexican children report a low frequency of PEB. These findings align with previous studies conducted in Latin America (Juárez-Lugo, 2014; Díaz-Marín and Geiger, 2019), in which participants reported low to medium PEB. There might be cultural reasons behind these results. For instance, Milfont and Schultz (2016) found that culture influences the relationship between humans and nature. This might, in turn, lead to differences in the way people behave toward the natural environment. The possible cultural differences behind children’s PEB require further attention.

As expected (H2), connection to nature seems to be partly responsible for the higher PEB found in children living in rural areas. The associations found suggest that the stronger the connection children feel with the natural world, the more likely they are to behave in a pro-environmental way. This result is also in consonance with the findings of previous studies (Olivos et al., 2013; Whitburn et al., 2019), suggesting that connection to nature is linked to PEB both in developed and developing countries.

In line with the pattern often described in previous studies, girls report being slightly more connected to nature than boys (Duarte et al., 2017). In addition, the current study adds to the literature on gender differences in pro-environmentalism by demonstrating variation in the relationship between connection to nature and PEB, being this association stronger for girls than for boys. The reasons for this may be that females are usually socialized to consider the needs of others (Dietz et al., 2002). This might imply that girls are socialized to show more altruistic values and helping behavior toward others, including nature and natural elements, than boys (McCright, 2010). This might, in turn, strengthen the link between girls’ emotional connection to nature (i.e., connection to nature) and their behavior (i.e., PEB). Close examinations of these possibilities remain for future studies.

Our findings point in the same direction as those of previous researchers (Otto and Pensini, 2017; Rosa et al., 2019), suggesting that contact with nature can be a way of promoting children’s pro-environmentalism. Other factors involved in experiences with nature should also be considered when trying to explain children’s PEB, such as the type of nature in which children spend their time (Collado et al., 2015) and the perception of aesthetic qualities in natural areas (Lumber et al., 2017). Living close to nature is not always possible and other ways of providing opportunities for children’s contact with nature should be considered. For example, schoolyards could play an important role in enhancing urban children’s time spent in natural areas (Amicone et al., 2018). Introducing nature in the classroom, such as wall gardens, can also be an effective way for children to experience nature in their daily live (van den Berg et al., 2017). Another strategy that can help to bring children close to nature is incorporating technology into the classroom. Presenting a video or images from natural areas has benefits such as increased positive emotions (Zelenski et al., 2015) and a sense of wellbeing (Capaldi et al., 2015). Being exposed to nature through videos/images in the classroom could be complementary to direct contact with nature.

Because environmental education programs have a stronger effect when conducted on young children than on adults (Liefländer and Bogner, 2014), and because most programs are aimed at children, we encourage the organization of environmental programs to be carried out in rural areas and include direct contact with nature (De Dominicis et al., 2017; Otto and Pensini, 2017). This might lead, in turn, to a stronger connection to nature and PEB. Given the differences found between urban/rural regarding their connection to nature and PEB, we believe that environmental education programs should be designed taking into consideration children’s place of residence and their frequency of contact with nature. We hope the findings of this cross-sectional study serve as an inspiration for testing out interventions, as this will most likely help establish a causal link between exposure to nature and pro-environmentalism.

Despite the contributions described above, some limitations should be noted when interpreting the results. First, this is a cross-sectional study and the effects found cannot be taken in a strictly causal sense. Nevertheless, our results are in line with previous studies highlighting the importance of the physical context (Hinds and Sparks, 2008; Collado et al., 2015) and connection to nature (Cheng and Monroe, 2012) when examining the factors leading to PEB. We believe they serve as a starting point for further experimental research. Second, it should be noted that the results of this brief research report only apply to a specific context: the Northern part of Mexico. The findings are consistent with previous studies conducted in developed countries (Evans et al., 2018), but further research in various developing countries is needed to generalize our results. A third limitation is that our study explains 45% of the variance from self-reported PEB. This percentage is similar to the one found in previous studies with children (Collado and Evans, 2019), but other variables such as social norms (Casaló and Escario, 2016) and perceived restorativeness (Collado and Corraliza, 2015) might help us obtain a deeper understanding of children’s PEB.

Limitations aside, from a theoretical point of view this study shows the relevance of place of the residence (urban/rural) for Mexican children’s PEB, as well as the mediating role of connection to nature in the place of residence–PEB relationship. We believe it is essential to expand the findings of this brief research report by studying the possible influence that living in places with a variety of physical characteristics (e.g., the beach, the mountains, and the city) can have in people’s PEB. Given that children will be the ones taking care of the natural environment in the near future, the inclusion of children from developing countries in such research seems essential.

## Data Availability Statement

The datasets generated for this study are available on request to the corresponding author.

## Ethics Statement

The studies involving human participants were reviewed and approved by the Comité de Ética Institucional del Instituto Tecnológico de Sonora. The patients/participants provided their written informed consent to participate in this study.

## Author Contributions

MD-R and FG-V conceived and designed the study. MB-E collected the data. MD-R, SC, and FG-V analyzed the data and wrote an initial draft based on the results. SC critically revised the draft manuscript and made important changes in the content.

## Conflict of Interest

The authors declare that the research was conducted in the absence of any commercial or financial relationships that could be construed as a potential conflict of interest.

## Footnotes

1 This specific model was chosen (compared to other models, such as number 59) because preliminary regression analyses suggested that gender only affected the relation between connection to nature and PEB.
